# Can SGLT2 inhibitors answer unmet therapeutic needs in chronic kidney disease?

**DOI:** 10.1007/s40620-022-01336-7

**Published:** 2022-05-18

**Authors:** Luca De Nicola, Mario Cozzolino, Simonetta Genovesi, Loreto Gesualdo, Giuseppe Grandaliano, Roberto Pontremoli

**Affiliations:** 1 Nephrology and Dialysis Unit, Department of Advanced Medical and Surgical Sciences, University Vanvitelli, Naples, Italy; 2 Renal Division, ASST Santi Paolo e Carlo, Department of Health Sciences, University of Milan, Milan, Italy; 3 School of Medicine and Surgery, Nephrology Clinic, University of Milano-Bicocca, Milan, Italy; 4 Istituto Auxologico Italiano, IRCCS, Milan, Italy; 5 Department of Emergency and Organ Transplantation (DETO), School of Medicine, University of Bari “Aldo Moro”, Bari, Italy; 6 Dipartimento di Medicina e Chirurgia Traslazionale, Università Cattolica del Sacro Cuore, Rome, Italy; 7 Dipartimento di Scienze Mediche e Chirurgiche, U.O.C. Nefrologia, Fondazione Policlinico Universitario A. Gemelli IRCCS, Rome, Italy; 8 Department of Internal Medicine, University of Genoa and IRCCS Ospedale Policlinico San Martino, Genoa, Italy

**Keywords:** SGLT2i, CDK, Diabetes, ESKD

## Abstract

Chronic kidney disease (CKD) is a global health problem, affecting more than 850 million people worldwide. The number of patients receiving renal replacement therapy (dialysis or renal transplantation) has increased over the years, and it has been estimated that the number of people receiving renal replacement therapy will more than double from 2.618 million in 2010 to 5.439 million in 2030, with wide differences among countries. The main focus of CKD treatment has now become preserving renal function rather than replacing it. This is possible, at least to some extent, through the optimal use of multifactorial therapy aimed at preventing end-stage kidney disease and cardiovascular events. Sodium/glucose cotransporter 2 inhibitors (SGLT2i) reduce glomerular hypertension and albuminuria with beneficial effects on progression of renal damage in both diabetic and non-diabetic CKD. SGLT2 inhibitors also show great benefits in cardiovascular protection, irrespective of diabetes. Therefore, the use of these drugs will likely be extended to the whole CKD population as a new standard of care.

**Graphical abstract:**

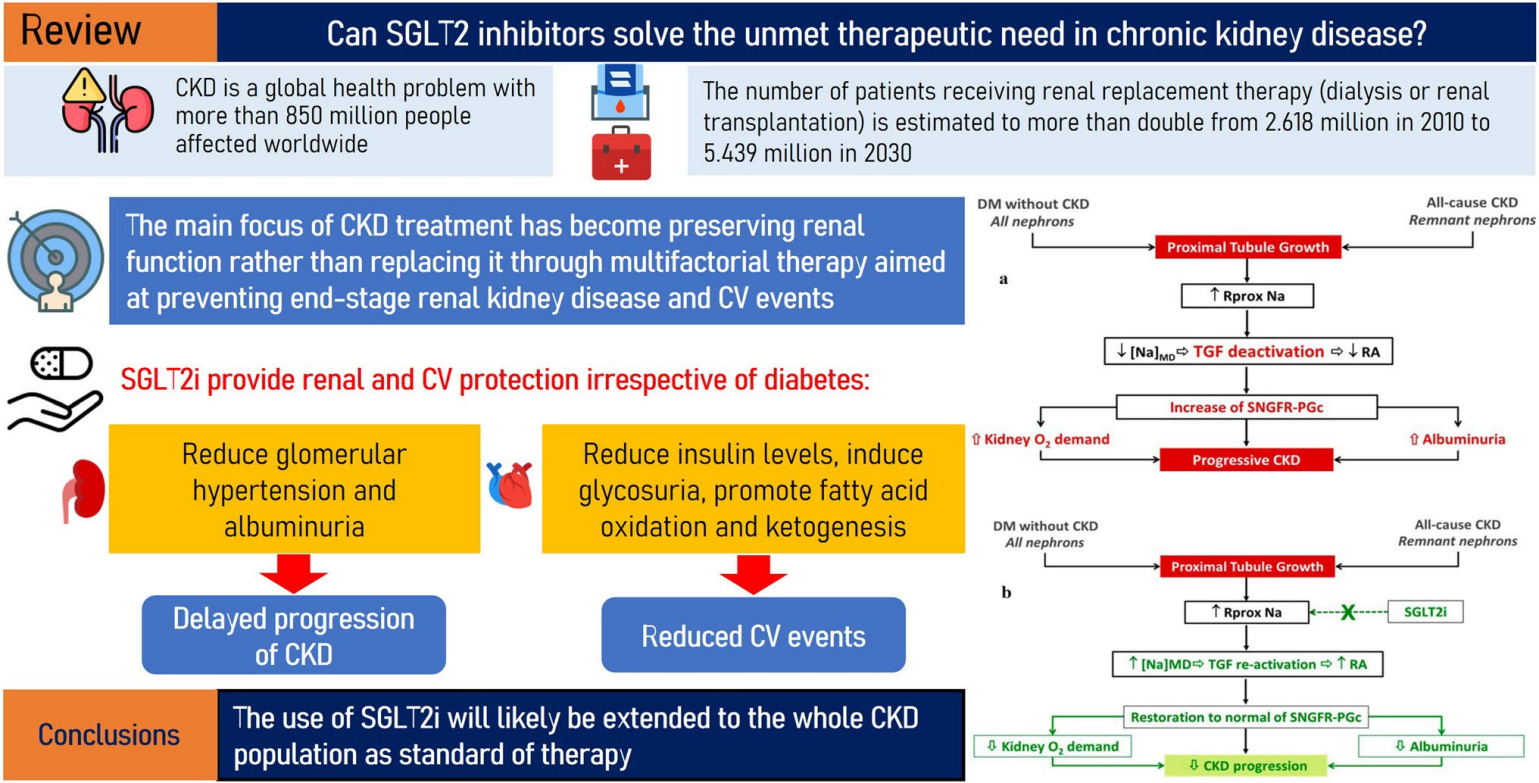

## Introduction

Chronic kidney disease (CKD) is a global health problem, affecting more than 850 million people worldwide [1]. CKD mortality rates increased 41.5% between 1990 and 2017, whereas mortality associated with ischemic heart disease, stroke, and chronic obstructive pulmonary disease decreased over the same period [2]. The need for renal replacement therapy (RRT) is projected to increase substantially over the next few decades [3]. Therefore, CKD is a major health problem in developed countries. Moreover, the cost of care is high, especially when prevention strategies are not implemented [4].

The Global Burden of Disease study has shown that, from 1990 to 2016, the incidence of CKD increased by 89% to 21,328,972, prevalence increased by 87% to 275,929,799, death due to CKD increased by 98% to 1,186,561, and disability-adjusted-life years increased by 62% to 35,032,384 worldwide mainly because of population growth and aging [5]. Similarly, the number of patients receiving RRT (dialysis or renal transplantation) has increased over the years, and it has been estimated that the number of people receiving RRT will more than double from 2.618 million people worldwide in 2010 to 5.439 million in 2030 [3]. A National Health Examination Survey in Italy evaluated the prevalence of CKD and the associated cardiovascular (CV) risk factors in the general Italian adult population. Results showed that the prevalence of CKD in Italy is relatively low when compared to other countries (7% in Italy vs 13% in the US), despite the older age and unfavorable CV risk profile of the whole population [6]. Indeed, CKD prevalence is heterogeneous across the different countries of world; the variability of CKD estimates is possibly due not only to methodological confounders but also to genetic and dietary determinants, that in the Mediterranean area may be protective against CKD onset as observed for cardiovascular events [7].

## Unmet needs in the management of CKD

Management of CKD has changed in the last 30 years. Thirty years ago, management was mainly focused on dialysis because medical (conservative) therapy was considered ineffective even for moderate degrees of CKD (serum creatinine level ≥ 1.5–2.0 mg/dL) [8]. However, knowledge on CKD pathophysiology has greatly improved in recent years and, in parallel, it has been observed that dialysis, albeit a life-saving treatment, does not improve the patient’s conditions or long-term survival. Therefore, the main focus of CKD treatment has now become preservation rather than replacement of renal function [9]. This is possible through the optimal use of multifactorial therapy aimed at preventing end-stage kidney disease (ESKD) and CV events [10, 11]. A key component of the multifactorial approach is antiproteinuric intervention, which is essential to improve patient and renal survival; in fact, several trials have proved that lowering albuminuria leads to better renal survival over the long term [12].

Unfortunately, however, management of CKD remains suboptimal because of numerous unmet needs. First, awareness of CKD is still low. Despite the increasing burden, CKD remains substantially underdiagnosed, particularly in the earlier stages where there is the greatest potential to slow progression [13–16].

In Italy, awareness of CKD is estimated to be overall low (less than 10% of patients affected by CKD), and even in the more advanced stages (less than 20% at CKD stages 3–5) [6]. This is mainly related to the low level of awareness among primary care physicians and specialists other than nephrologists, who do not identify CKD using two simple and cheap diagnostic tools: serum creatinine to estimate GFR and urine albumin tests. Increasing awareness of CKD is one of the main goals of the nephrology community [6].

An important problem related to low awareness is the late referral of patients with CKD to nephrologists. It has been shown that early referral leads to a reduction in mortality and hospitalization, whereas unreferred patients have a higher risk of developing ESKD and a higher mortality rate [17–20]. On the other hand, the PIRP (Prevenzione Insufficienza Renale Progressiva) study conducted in Emilia Romagna (Italy) demonstrated that active communication between general practitioners (GPs) and nephrologists, allowing integrated management of the disease, led to the establishment of early treatment with a consequent reduction in the rate of dialysis among patients with CKD participating in the study [21].

An additional but relevant barrier to efficacious treatment of CKD is the suboptimal effectiveness of current nephroprotective agents. In the last decades, many randomized clinical trials (RCTs) testing new therapies have failed to demonstrate efficacy and have raised concerns on treatment-related adverse effects. On the other hand, sodium/glucose cotransporter 2 inhibitors (SGLT2i) and atrasentan have shown positive effects. The positive results obtained by atrasentan have not translated to marketing of the drug for use in clinical practice due to the reduced interest by the producer related to expiration of the atrasentan patent [22]. Therefore, renin–angiotensin–aldosterone system (RAAS) inhibitors remain the first-choice treatment to prevent progression of CKD; however, their use is limited by the risk of hyperkalemia and acute kidney injury [23]. Furthermore, RAAS inhibitors are characterized by a high residual cardio-renal risk [22]. Therefore, it is not surprising that the use of RAAS inhibitors in the CKD population after a positive trend from 1999 to 2010 did not increase further in the following decade [24].

These concepts have recently been highlighted by the new guidelines on diabetic kidney disease (DKD), which recommend treatment with angiotensin-converting enzyme (ACE) inhibitors or angiotensin II receptor blockers (ARBs) for CKD patientswith diabetes and albuminuria. According to the guidelines, these medications must be titrated to the highest approved tolerated dose and every effort should be made to maintain this therapy [25]. Nevertheless, as stated in the “practice points” section, patients treated with RAAS inhibitors must be carefully monitored to prevent (or promptly treat) the main and most common side effects; that is, hyperkalemia and acute kidney injury.

Finally, large gaps exist in the  care of non-diabetic CKD patients [26]. More CKD patients with diabetes versus those without diabetes had their albumin-to-creatinine ratio (ACR) assessed (64.2 vs. 17.0%) or received an ACE inhibitor/ARB (78.3 vs. 58.1%) or statins (64.6 vs. 39.2%). Nevertheless, the residual cardiorenal risk of diabetic CKD remains high, even in patients receiving intensive multifactorial medical therapy [27].

## The call for a CKD screening program

The cost of CKD care is high, especially when prevention strategies are not implemented [4]. In the United States, Medicare costs for CKD patients aged 65 years and older exceeded US$81 billion in 2018, representing 22% of all Medicare spending in this age group. More than 70% of Medicare spending for these patients was incurred by those who also had diabetes, congestive heart failure, or both. Moreover, pro capita spending was more than twice as high for patients with all three chronic conditions, i.e., CKD, diabetes, and congestive heart failure (US$ 57,965) than for patients with CKD alone (US$ 25,734). Also, total Medicare-related expenditure for beneficiaries with ESKD increased to US$ 49.2B in 2018, accounting for 7.2% of Medicare fee-for-service expenditure [28].

Elshahat and colleagues [29] estimated the mean annual health care cost per patient with CKD in high income countries. The results showed that progression from CKD G1-G2 to CKD G3a-3b was associated with a 1.1- to 1.7-fold increase in the mean annual health care cost per patient [29].

CKD is a public health problem, which involves not only nephrologists but also GPs and numerous non-nephrologist clinicians and diabetologists [30]. It has been shown in fact that, in addition to ESKD, less severe kidney dysfunction also has an extraordinarily high negative impact on the incidence of fatal and nonfatal cardiovascular events [31]. The relative risks of mortality and ESKD by estimated glomerular filtration rate (eGFR) and albuminuria are the same, irrespective of the presence or absence of diabetes, hypertension and independently of the age of patients as well [32].

For the reasons mentioned above, early identification of CKD by screening for kidney disease, followed by risk stratification and treatment, has the potential to substantially reduce the morbidity and mortality of CKD and its related complications, such as CV disease, as well as the costs related to the management of advanced CKD. However, there is no accepted systematic strategy for early detection and treatment of CKD.

Despite effective methods to diagnose and treat CKD in its earliest stages, there is a lack of consensus on whether health systems and governments should implement CKD screening programs. Professional associations have been discordant on whether or not to screen for CKD [33]. In particular, an effective and efficient approach could prevent missing patients who need to be followed and wasting resources on unnecessary consultations.

To address this ongoing controversy, in October 2019, the Kidney Disease: Improving Global Outcomes (KDIGO) organization held a Controversies Conference entitled “*Early Identification and Intervention in CKD*”. Three themes mainly related to the patients were highlighted during the conference as being important underlying principles for CKD screening strategies: patients overwhelmingly prefer earlier CKD screening and diagnosis; patient education has the potential to improve self-management and disease prognosis; economic rationale must favor some program of early CKD screening/risk stratification/treatment, given the costs of kidney failure to health care systems and society [25]. The conference participants therefore concluded that the decisions concerning the age to initiate testing, the frequency of repeat testing, and the time to forgo or end testing should all be  tailored based on risk factors, patient preferences, and life expectancy [25].

Efforts for the early detection of CKD should first be implemented in individuals with established CKD risk factors, mainly hypertension, diabetes and CV disease, given the higher expected prevalence of CKD among these individuals. Identifying and treating all cases of CKD would be the most complete approach to improving kidney health and reducing the burden of kidney disease. However, population-wide CKD screening  programs are known to have potential drawbacks, including higher costs and greater barriers to implementation than targeted high-risk screening. CKD screening and treatment programs should also be implemented in other high-risk individuals and populations based on comorbidities, environmental exposures, or genetic factors. CKD screening for persons with these risk factors should be guided by tailored clinical assessment and joint decision making, rather than a uniform approach. In these patients, the initiation, frequency, and cessation of CKD screening should be tailored based on kidney and CV risk profiles and individual preferences. Moreover, the timing and frequency of the screening should be tailored based on the patient’s characteristics:  the point in life when screening should begin should be based on the estimated likelihood of that individual having CKD, rather than on age alone; the frequency of repeat testing should not be uniform for all persons, but rather should be guided by each individual’s risk of developing CKD, based in part on the results of previous testing [25].

The diagnosis of CKD among older adults is currently a controversial topic, as they experience the greatest burden of CKD and are at the highest risk for certain complications, such as CV disease and ESKD. There is concern that CKD is overdiagnosed among older adults and there have been calls for an age-adapted definition. However, underdiagnosis of CKD in older adults also carries consequences because older adults have the highest prevalence of CKD, and CKD has an impact on their physical and cognitive functions, medication safety, and CV prognosis. Although there is potential harm associated with overdiagnosis of CKD in older adults, CKD should be properly diagnosed and risk stratified in older adults using all available measurements, including cystatin C testing in those with a serum creatinine-based eGFR of 45–59 mL/min per 1.73 m^2^ and ACR < 30 mg/g [34].

For the reasons mentioned above, CKD screening and risk stratification must consist of an assessment of both eGFR and urinary albumin-creatinine ratio (UACR). However, despite guideline recommendations, many clinicians currently fail to assess albuminuria in patients with reduced eGFR or diabetes [35].

## The role of primary care: from diagnosis to referral

Early diagnosis is crucial to prevent progression of CKD. GPs are the starting point in CKD patient care, therefore they require some skills to guarantee the best management of the disease by choosing the right treatment and avoiding progression, complications, and dialysis. Serum creatinine levels should be carefully evaluated, and values should be interpreted according to the patient’s muscle mass, age, limb amputation, and height [36]. GPs should be aware that some medications (such as certain antibiotics) might increase serum creatinine levels. For this reason, blood urea nitrogen (BUN) levels should always be measured to  identify the true increase in serum creatinine. BUN never changes when the increase is due to antibiotic treatment [37]; however, an increase in azotemia that is not proportional to renal function impairment occurs in dehydration and depletion conditions.

Extensive evaluation of patients with a decrease in GFR and proteinuria is suggested in all cases. A persistent GFR of < 60 mL/min per 1.73 m^2^, as well as persistent proteinuria predict a poor outcome of CKD [38]. Thus, quantitative and precise methods are always preferred over semiquantitative methods [38].

Frequent follow-up is fundamental for patients with early-stage CKD to slow progression and avoid complications. In this scenario, some nephrotoxic medications (such as nonsteroidal anti-inflammatory drugs, aminoglycoside antibiotics, and radiocontrast agents [38] or phosphate-containing bowel preparations [39]) should be avoided; oral preparations containing magnesium or aluminum should also be avoided. Blood pressure should be carefully monitored, aiming to keep it under 130/80 mm Hg. Moreover, urine protein to creatinine ratio should be assessed periodically [38]. According to the most recent guidelines [40], a systolic blood pressure target of less than 120 mm Hg is recommended in most patients with CKD [40]. ACE inhibitors and ARBs should be used to prevent worsening of albuminuria and the decline in GFR [41–43].

**Fig. 1:**
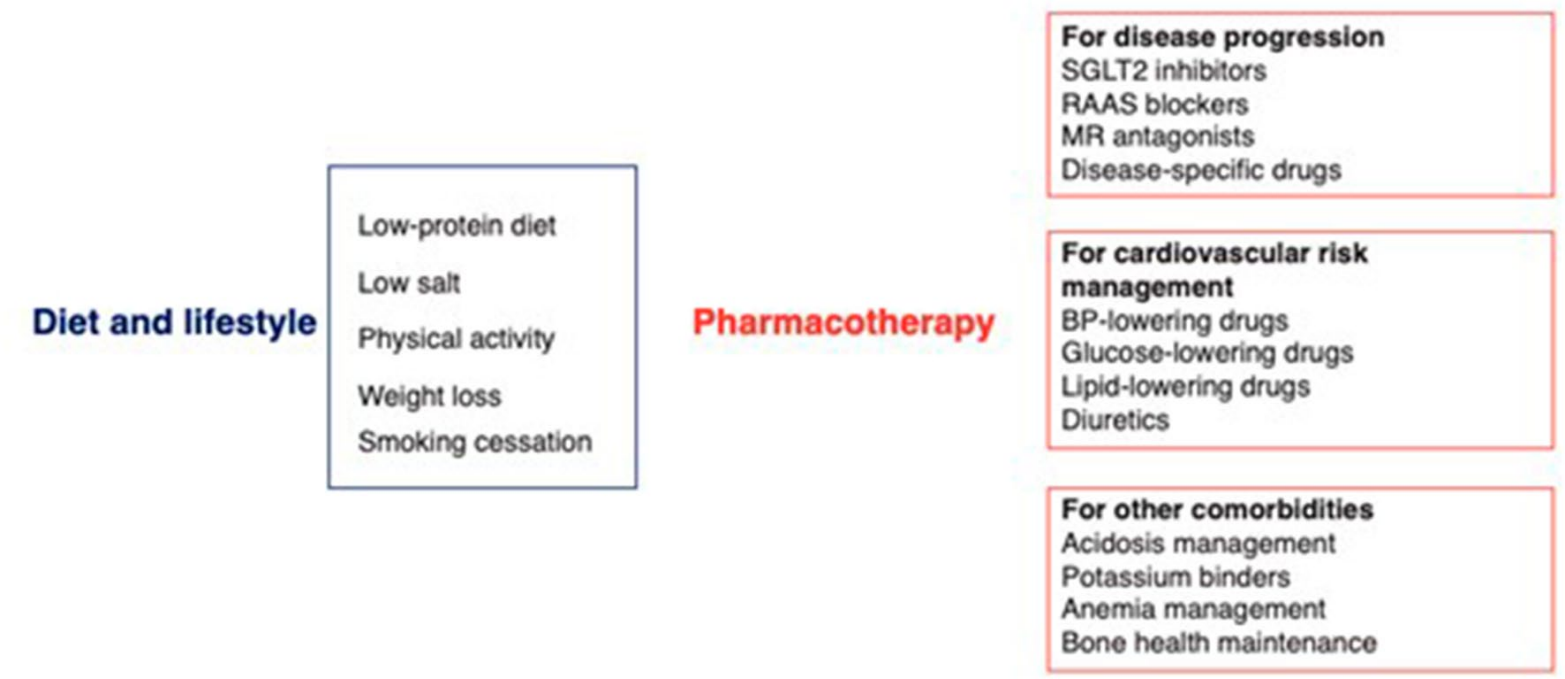
Kidney-preserving care. *BP* blood pressure, *MR* mineralocorticoid receptor, *RAAS* renin–angiotensin–aldosterone system, *SGLT2* sodium/glucose cotransporter 2. Adapted from [44]

Patients with CKD who have anemia should be treated with erythrocyte-stimulating agents. For these patients, an evaluation to assess iron deficiency or vitamin deficiencies should always be performed, as should routine tests, including reticulocyte count and measurement of serum vitamin B12 and folate, serum iron, ferritin, and total iron-binding capacity [38]. Finally, in patients with recurrent stone disease, an in-depth metabolic evaluation should be performed with the aim of identifying and treating modifiable risk factors, preventing further episodes, and promoting stone dissolution [38]. A therapeutic approach aimed at preserving kidney function is shown in Fig. 1 [44]. In conclusion, better communication between GPs and nephrologists, together with early treatment, can have a positive effect on the outcome of CKD and improve nephroprotection.

## Nephroprotection by SGLT2 inhibitors

Kidney diseases lead to a reduction in kidney mass, which is associated with single nephron hyperfiltration in the surviving nephrons, the so-called “remnant nephron hypothesis”. Single nephron hyperfiltration is the result of an increase in glomerular capillary pressure and albuminuria, which in turn induces tubular-interstitial inflammation [45]. Glomerular hypertension, caused by increased efferent arteriole vasoconstriction (mediated by angiotensin II) and/or increased afferent arteriole vasodilation (caused by deactivation of tubulo-glomerular feedback), can directly cause glomerular damage. Progression of renal damage is delayed by inhibition of the RAAS mediated by a reduction in glomerular capillary pressure and related reduction in albuminuria [46, 47]. Similar mechanisms occur in all nephrons of diabetic patients since the early stages of disease [47].

**Fig. 2:**
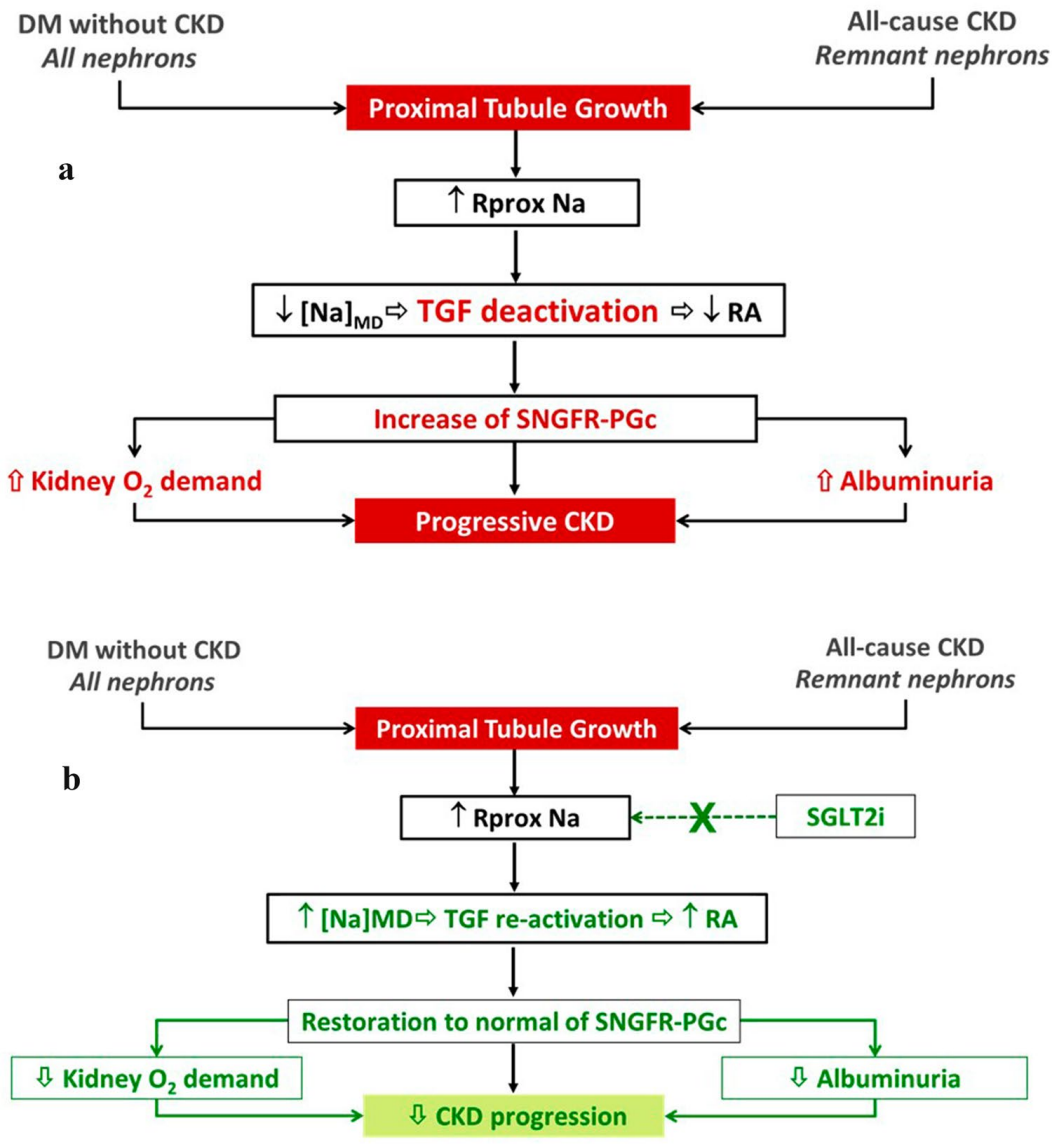
Hyperfiltration as a major determinant of CKD progression: role of SGLT2i. Hyperfiltration in the absence of (**A**) or during (**B**) treatment with SGLT2i. Adapted from [47]

SGLT2 inhibitors can modulate activation of the tubulo-glomerular feedback through an increase in sodium delivery to the macula densa. The result is a reduction in the vasodilation of afferent arterioles and, in turn, reductions in glomerular hypertension and subsequent albuminuria with beneficial effects on the progression of renal damage. Hyperfiltration is indeed a major determinant of CKD progression modulated by SGLT2i (Fig. 2) [47].

SGLT2 inhibitors also show great benefits in CV protection. First, SGLT2 inhibitors are able to reduce insulin levels, induce glycosuria, and simultaneously promote fatty acid oxidation and ketogenesis. SGLT2 inhibitors act by upregulating AMPK and SIRT1, as well as by inhibiting mTOR. This downregulation of mTOR signaling results in different cellular repair mechanisms, which induce cellular stress resistance and diminish cellular senescence. This modulation improves the outcomes of metabolic diseases and attenuates vascular inflammation and arterial stiffness, thus increasing protection against oxidative stress [48].

Albuminuria represents a marker of the worsening of CV outcomes. Fitchett et al. [49] reported that empagliflozin was able to reduce albuminuria in patients who did not show any CV or renal outcome. Inflammatory pathways have been known to be involved in CV risk. These processes include activation of inflammasomes or the release of some proinflammatory cytokines. According to Kim et al. [50], empagliflozin was able to reduce the release of proinflammatory cytokines, such as interleukin 1B. At the same time, ketone production may increase due to the establishment of glycosuria by SGLT2 inhibitors. In particular, it has been shown that ketone production might be involved in the establishment of several anti-inflammatory and anti-oxidative mechanisms, which can improve CV outcomes [51].

Recently, SGLT2 inhibitors have been shown to increase levels of bone marrow-derived hematopoietic cells and to direct them to the vascular injury, thereby improving CV outcomes. Albiero et al. [52] conducted a study using an animal model of mice with streptozotocin-induced diabetes treated with dapagliflozin. Dapagliflozin was found to reduce glucose levels by 20% and to improve the defect in hematopoietic cell mobilization [52]. Jongs and colleagues [53] conducted a sub-analysis that evaluated the effects of dapagliflozin on albuminuria in CKD patientswith and without type 2 diabetes in the DAPA CKD trial. The results showed that dapagliflozin significantly decreased albuminuria by 35.1% and 14.8% in CKD patients with and without type 2 diabetes, respectively.

## Cardiovascular protection by SGLT2-I in patients with CKD

The original aim of SGLT2 inhibitors was to lower blood glucose levels in patients with type 2 diabetes. Numerous RCTs evaluating the effects of SGLT2 inhibitors on improving CV outcomes in patients have mainly focused on atherosclerotic CV disease (ASCVD)-related outcomes. In most trials, a risk reduction for major adverse cardiovascular events (MACEs), i.e., myocardial infarction, stroke, or CV death, was demonstrated [54]. Some of these trials were performed exclusively on patients with CKD, whereas in other trials, the design included enrolling patients with renal failure and patients with preserved renal function.

The CREDENCE study was the first double-blind randomized trial designed to evaluate the effect of canagliflozin on renal outcomes in people with type 2 diabetes and eGFR of 30 to < 90 mL/min and albuminuria. The primary outcome was a composite of ESKD, a doubling of serum creatinine level, or death from renal or cardiovascular causes; the secondary outcomes were a renal-specific composite (ESKD, doubling of serum creatinine, or renal death), a composite of CV death or hospitalization for heart failure, all-cause mortality, a composite of CV death, myocardial infarction, or stroke. The study involved 4,401 patients with type 2 diabetes and albuminuric kidney disease who were randomized to receive canagliflozin (100 mg daily) or placebo. The results showed a decrease in the risk of kidney and CV events (but not in all-cause mortality) in patients treated with canagliflozin compared to the placebo group [55].

The VERTIS CV trials randomly assigned 8246 patients with T2D and atherosclerotic cardiovascular disease to receive two doses of ertugliflozin or placebo. The primary outcome was the noninferiority of ertugliflozin to placebo with respect to a composite CV end-point (death from CV causes, nonfatal myocardial infarction, or nonfatal stroke). Altogether, 1199 patients who were randomized to Ertuglifozin and 608 who were randomized to placebo had an eGFR < 60 ml/min. The study showed that among patients with type 2 diabetes and atherosclerotic cardiovascular disease, ertugliflozin was noninferior to placebo with respect to major adverse cardiovascular events (HR 0.88; 95.8% CI 0.75–1.03; *P* = 0.11 for superiority). In the subgroup of patients with CKD, the results were similar (HR 1.08; 95.8% CI 0.84–1.40) [56].

DECLARE-TIMI 58 was designed to evaluate the effect of dapagliflozin on CV outcomes in patients with type 2 diabetes. Participants were randomized to receive dapagliflozin or placebo. The primary outcome was a composite of MACE and death or hospitalization for heart failure. Secondary outcomes were a renal composite (which included ≥ 40% decrease in eGFR to < 60 mL/min per 1.73 m^2^ of body surface area, new onset ESKD, or death from renal or CV causes) and death from any cause. The results showed a decreased rate of CV death or hospitalization in patients treated with dapagliflozin compared to those treated with placebo [57]. DECLARE-TIMI 58 CKD consisted of a secondary analysis of DECLARE-TIMI 58. The study aimed to evaluate the effect of dapagliflozin on CV outcomes in patients with type 2 diabetes and eGFR < 60 mL/min and/or albuminuria (UACR > 30 mg/g); 1265 patients with eGFR < 60 mL/min, 5199 patients with UACR > 30 mg/g, and 548 patients with both conditions were enrolled. The results showed that the effect of dapagliflozin on the relative risk for CV events was consistent across the eGFR and UACR groups, with the greatest absolute benefit for the composite of CV death or hospitalization for heart failure observed among patients with both reduced eGFR and albuminuria [58].

The EMPEROR Reduced trial was designed to evaluate the effect of empagliflozin on CV outcomes and mortality in people with heart failure with a reduced ventricular ejection fraction (HFrEF), with or without type 2 diabetes. Moreover, 48% of patients had eGFR < 60 mL/min. Patients were randomized to receive empagliflozin (10 mg once daily) or placebo, as well as the standard of care. The primary outcome was designed as a composite of CV death or hospitalization for worsening heart failure. Patients treated with empagliflozin presented a lower risk of CV death or hospitalization for heart failure than those in the placebo group, regardless of the presence or absence of type 2 diabetes [59].

DAPA HF was a phase 3 study that evaluated the effect of dapagliflozin on CV outcomes and mortality in people with HFrEF, with or without type 2 diabetes. Forty-one percent of patients had eGFR < 60 mL/min; 4744 participants were randomly assigned to receive dapagliflozin (10 mg once daily) or placebo and the composite of worsening heart failure or CV death was analyzed as the primary outcome. The results showed that the risk of worsening heart failure or death from CV causes was lower among the patients enrolled in the treatment group compared to those in the placebo group, regardless of the presence or absence of type 2 diabetes [60].

The SOLOIST WHF trial was designed to evaluate the efficacy and safety of SGLT2 inhibitors after an episode of decompensated heart failure. It was a multicenter, double-blind trial that enrolled 1222 patients randomized to receive sotagliflozin or placebo with follow-up for a median of 9 months. The primary outcome was the total number of deaths from CV causes, hospitalizations, and urgent visits due to heart failure. The results showed the efficacy of sotagliflozin in decreasing deaths, hospitalizations, and urgent visits due to CV causes [61]. The study showed that the protective effect of SGLT2 inhibitors on the CV system was stronger in patients with CKD than in those with preserved renal function.

DAPA CKD was a double-blind, placebo-controlled, phase 3 study that assessed whether treatment with dapagliflozin, compared to placebo, reduced the risk of renal and CV events in CKD patients with or without type 2 diabetes. The primary outcome included a composite of CV and renal outcome, such as a sustained decrease ≥ 50% in eGFR, ESKD, renal or CV death. The secondary outcome was CV death or hospitalization for heart failure. The results showed that the effects of dapagliflozin were similar in participants with and without type 2 diabetes. Moreover, no differences were found between patients with eGFR > 45 mL/min or < 45 mL/min, or between patients with proteinuria > 1000 mg/day or ≤ 1000 mg/day [62].

Recently, a prespecified subgroup analysis was conducted to evaluate the effects of dapagliflozin on kidney, CV, and mortality outcomes according to the presence or absence of type 2 diabetes and according to the underlying cause of CKD, such as diabetic nephropathy, chronic glomerulonephritis, ischemic or hypertensive CKD, or from other or unknown causes. A total of 386 study sites in 21 countries were included in the analysis. The results showed a reduction in major adverse kidney and CV events, as well as in all-cause mortality in CKD patients, with or without diabetes [63]. A post-hoc analysis of the trial showed that patients with stage 4 CKD randomized to dapagliflozin experienced a significant reduction in the primary composite endpoint and in CV and mortality endpoints compared to placebo. No interaction was present when comparing CKD stage 4 versus stages 2/3 [64].

The results from the EMPEROR-Preserved trial were published recently. This was a double-blind study that included 5,988 patients with class II–IV heart failure and an ejection fraction of more than 40%. Patients were randomly assigned to receive empagliflozin (10 mg once daily) or placebo, in addition to their usual therapy. The aim of the study was to evaluate the effects of empagliflozin on major heart failure outcomes in patients with heart failure with preserved ejection fraction [65]. The results showed that, over a median of 26.2 months, a primary outcome event (a composite of CV death or hospitalization for heart failure) occurred in 13.8% of patients in the empagliflozin group and in 17.1% in the placebo group (95% confidence interval 0.69–0.90; *P* < 0.001), regardless of the presence of diabetes [65]. A lower number of hospitalizations due to heart failure were reported in the treatment group compared to the placebo group [64]. Interestingly, the same trial demonstrated halving of the rate of eGFR decline in the experimental arm.

The clinical studies mentioned above are summarized in Table 1. Another study, EMPA-Kidney (NCT03594110), has been started recently. This is an ongoing RCT of empagliflozin versus placebo in people with CKD, with or without type 2 diabetes. EMPA-Kidney will assess whether empagliflozin reduces the risk of kidney disease progression or CV death.

**Table 1:** Major SGLT2i trials on cardiovascular outcomes in chronic kidney disease patients

Study	Aim	Patients	Drug	Results
CREDENCE [55] VERTIS CV [56]	To evaluate the effect of canagliflozin on renal outcomes in people with T2D and eGFR between 30 and < 90 mL/min and albuminuria To evaluate the noninferiority of ertugliflozin to placebo with respect to a composite CV end-point	4401 patients with T2D and albuminuric kidney disease 8246 patients with T2D and atherosclerotic cardiovascular disease	Canagliflozin Ertuglifozin	Decreased risk of renal and CV events (but not of all-cause mortality) in patients treated with canagliflozin compared to the placebo group Noninferiority of ertugliflozin to placebo
DECLARE-TIMI 58 [57]	To evaluate the effect of dapagliflozin on CV outcomes in patients with T2D	17,160 patients with T2D and high CV risk	Dapagliflozin	Decreased rate of CV death or hospitalization in patients treated with dapagliflozin compared to the placebo group
DECLARE-TIMI58 CKD [58]	To evaluate the effect of dapagliflozin on cardiovascular outcomes in patients with T2D and eGFR < 60 mL/min and/or albuminuria (UACR > 30 mg/g)	1265 patients with an eGFR < 60 mL/min, 5199 patients with a UACR > 30 mg/g and 548 patients with both conditions	Dapagliflozin	The effect of dapagliflozin on the relative risk for CV events was consistent across eGFR and UACR groups, with the greatest absolute benefit for the composite of CV death or hospitalization for HF observed among patients with both reduced eGFR and albuminuria
EMPEROR Reduce Trial [59]	To evaluate the effect of empagliflozin on CV outcomes and mortality in people with HFrEF, with or without T2D	1863 patients with chronic HF (functional class II, III, or IV) with left ventricular ejection fraction of 40% or less	Empagliflozin	Patients treated with empagliflozin presented a lower risk of cardiovascular death or hospitalization for heart failure than those in the placebo group, regardless of the presence or absence of diabetes
DAPA HF [60]	To evaluate the effect of dapagliflozin on CV outcomes and mortality in people with HFrEF, with or without T2D	4744 patients with an ejection fraction of 40% or less, class II, III, or IV symptoms, and plasma level of N-terminal pro–B-type natriuretic peptide (NT-proBNP) of at least 600 pg/mL	Dapagliflozin	The risk of worsening HF or death from CV causes was lower among the patients enrolled in the treatment group compared to the placebo group, regardless of the presence or absence of T2D
SOLOIST WHF [61]	To evaluate the efficacy and the safety of SGLT2 inhibitors after an episode of decompensated HF	1,222 patients with T2D who had been hospitalized for HF, and who received treatment with intravenous diuretic therapy	Sotagliflozin	The protective effect of the SGLT2 inhibitor on the cardiovascular system is stronger in patients with CKD than in those with preserved renal function
DAPA CKD [62]	To evaluate whether treatment with dapagliflozin, compared to placebo, reduced the risk of renal and CV events in patients with CKD with or without T2D	4304 CKD patients with or without T2D	Dapagliflozin	The effects of dapagliflozin were similar in participants with and without T2D. Moreover, no differences were found between patients with eGFR > or < 45 mL/min, or between patients with proteinuria > or < 1000 mg/day. The effects of dapagliflozin were not different in CKD stage 4 patients compared to CKD stage 2/3
EMPEROR-Preserved [65]	To evaluate the effects of empagliflozin on major HF outcomes in patients with HF and preserved ejection fraction	5988 patients with class II–IV HF and an ejection fraction of more than 40%	Empagliflozin	Reduction of the combined risk of CV death or hospitalization for HF in patients treated with empagliflozin with HF and preserved ejection fraction, regardless of the presence or absence of diabetes

*CKD* chronic kidney disease, *CV* cardiovascular, *eGFR* estimated glomerular filtration rate, *HF* heart failure, *HFrEF*, heart failure with a reduced ventricular ejection fraction, *SGLT2* sodium/glucose cotransporter 2, *T2D* type 2 diabetes, *UACR* urine albumin-creatinine ratio

In general, some heterogeneity across the different classes of SGLT2 inhibitors for selected outcomes has been found (especially for MACE and CV death), which requires further exploration. On the other hand, other CV benefits, such as reduction in hospitalization risk for heart failure, showed only moderate heterogeneity among the drugs [54].

## SGLT2 inhibitors: their place in therapy

Blood pressure reduction, glucose lowering, and RAAS inhibition have shown limited effectiveness in the prevention and treatment of DKD. Accordingly, there has been only modest improvement in the prevention of ESKD over the last 2–3 decades (Table 2) [66]. For this reason, aggressive multifactorial therapy is recommended for the treatment of CKD in patients with diabetes.

**Table 2:** Trend in the incidence of end-stage kidney disease in the United States over the last decades

Year	Diabetes, *n*	End-stage kidney disease, *n*
1990	6,536,163	17,763
1995	7,862,661	29,259
2000	11,799,201	41,477
2005	16,066,108	46,917
2010	20,676,427	50,197

A lack of improvement in the prevention of end-stage kidney disease over time is evident. Adapted from [54]

RAAS inhibitors are an important element in the treatment of DKD, and have been shown to delay the progression of the disease in patients with proteinuria > 300 mg/day. However, efficacy is not the same among patients with diabetes. A paradoxical relationship between blood pressure reduction and renal morbidity (the so-called J curve) may reduce the benefit of the treatment, and especially so in patients with minimal or no albuminuria [67]. It has been shown that a very low blood pressure level can paradoxically be associated with an increase in renal morbidity. For this reason, numerous reports suggest a systolic target of 120 to 130 mm Hg (but not < 120 mmHg) to improve both renal and CV outcome [68]. SGLT2 inhibitors have demonstrated an important beneficial additive effect with RAAS inhibitors, regardless of the presence of albuminuria and the GFR [67]. Several RCTs have reported both CV and renal protection in patients with DKD [55–57, 69, 70] (Table 3).

**Table 3:** Renal protection in patients with diabetic kidney disease in the most important Cardiovascular Outcome Trials with SGLT2 inhibitors

Trial	EMPA REG OUTCOME (Barutta)	CANVAS PROGRAM (Barutta)	DECLARE-TIMI 58 (Wiwiott)	VERTIS CV (Cosentino)
Kidney composite outcomes	Sustained ≥ 40% reduction in eGFR, renal replacement therapy (dialysis or transplantation), or death from renal causes	Sustained ≥ 40% reduction in eGFR, renal replacement therapy (dialysis or transplantation), or death from renal causes	Sustained ≥ 40% decrease in eGFR to < 60/mL/min per 1.73 m^2^ and/or end-stage renal disease and/or renal death	Sustained ≥ 40% reduction in eGFR, renal replacement therapy (dialysis or transplantation), or death from renal causes
Hazard ratio (95% confidence interval)	0.55 (0.41, 0.73)	0.60 (0.47, 0.77)	0.53 (0.43, 0.66)	0.66 (0.50, 088)

*eGFR* Estimated glomerular filtration rate

A meta-analysis of available Cardiovascular Outcome Trials suggests that renal protection is maintained even when eGFR is < 60 mL/min per 1.73 m^2^. However, glycosuria and the glucose-lowering effect of SGLT2is are greatly reduced when GFR is below 45 ml/min. Thus, renal protection by SGLT2 inhibitors seems to be independent of the urinary excretion of glucose as well as of the glucose-lowering effect [71].

In conclusion, SGLT2 inhibitors have rapidly become the standard of care for DKD to delay progression to ESKD, even when a reduction in GFR limits their antihyperglycemic effect. Although this class of drugs has only been tested on top of RAAS inhibitors (and not in head-to-head comparisons), by indirect comparison it appears that SGLT2 inhibitors have a much greater renal protective potential compared to ACE inhibitors and ARBs (about 40% reduced rank regression (RRR) compared to 15–18%). Furthermore, RAAS inhibitors are not for all patients as they are less effective in the non-albuminuric phenotype and may worsen GFR in ischemic nephropathy. Conversely, SGLT2 inhibitors show a better safety profile than RAAS inhibitors.

Moreover, a prespecified analysis of the DAPA CKD trial aimed to evaluate the effects of dapagliflozin on kidney, CV, and mortality outcomes, regardless of the presence or absence of type 2 diabetes. The study, which involved more than 4,000 patients (2,152 patients randomized to receive dapagliflozin and 2,152 patients receiving placebo), showed a reduction in the risk of major adverse kidney and CV events and all-cause mortality in patients with diabetic and non-diabetic CKD [63]. Interestingly, a recent prespecified pooled analysis of DAPA-HF and DAPA-CKD trials revealed a significant 33% reduction in the incidence of new-onset type 2 diabetes in dapagliflozin-treated non-diabetic patients [72]. These exciting results, that were observed in the non-diabetic subgroup of the DAPA CKD trials, are very promising and support the use of dapagliflozin even in patients without diabetes.

This new vision of nephroprotective therapy encompasses the need to shift from testing a single intervention to evaluating the effectiveness of multiple interventions under the umbrella of “personalized medicine”. It is in fact growing the hypothesis of gaining better renal and patient outcomes when SGLT2 inhibitors are combined with low protein diets [73, 74], that as SGLT2 and RAAS inhibitors reduce hyperfiltration, as with new nephroprotective agents, namely Endothelin A Receptor Antagonists (ERA) and nonsteroidal mineral receptor antagonist (MRA) [75, 76]. In particular, recent data comparing the FIDELIO and CREDENCE trials have shown that the nephroprotective effects of finerenone are similar to those of canagliflozin when tested in similar patient populations [77, 78]. An intriguing clinical question is whether the addition of finerenone in patients who are already taking SGLT2i can provide additional benefits in terms of CV and renal protection, and whether the association can be considered safe. Few data are available in this regard. A recent sub-analysis of the FIDELIO-CKD, performed in 259 (4.6%) out of 5,674 patients taking an SGLT2i at baseline, showed a significant reduction in urine albumin-to-creatinine ratio and in renal and cardiovascular outcomes versus placebo, irrespective of the use of SGLT2i at baseline. In addition, patients taking both drugs had a lower incidence of hyperkalemia (8.1% with SGLT2i use versus 18.7% without SGLT2i use at baseline) [79].

It is worth noting that the guidelines of of the main scientific associations have recognized the importance of using this class of drugs in diabetic patients with CV diseases and/or CKD. The 2019 European Society of Cardiology Guidelines [80] recommend SGLT2i as a first-line treatment in patients with T2D in order to reduce mortality and CV events. The 2020 Kidney Disease Improving Global Outcomes Clinical Practice Guidelines [81] also give a first-line therapy indication for SGLT2i in all diabetic patients with eGFR > 30 ml/min, while the most recent American Diabetes Association Guidelines [82] recommend the use of this class of drugs in all patients at high risk of atherosclerosis, CV disease, heart failure, and/or CKD.

## Conclusions

CKD management has changed drastically during the last 30 years, shifting the focus from renal replacement therapy to preservation of renal function. This is possible through the optimal use of multifactorial therapy, which aims to prevent ESKD and reduce the risk of a CV event. Unfortunately, numerous unmet needs are involved in the non-optimal management of the disease. First, there is little awareness of CKD, together with the related problem of the late referral of patients with CKD to nephrologists.

On the other hand, early identification of CKD by screening for kidney disease, followed by risk stratification and treatment, offers the potential to substantially reduce the morbidity and mortality of CKD, its related complications, and the high cost of dealing with advanced CKD. Efforts for early CKD detection should focus on individuals with established CKD risk factors. Because GPs are the first to be involved in the care of patients with CKD, they need to acquire some skills to guarantee the best management of the disease, choosing the right treatment to avoid CKD progression, complications, and dialysis. In addition, better communication between GPs and nephrologists, together with early treatment could positively affect the outcome of CKD and improve nephroprotection.

Finally, the suboptimal effectiveness of current nephroprotective agents (which still carry a significant residual risk of progression to ESKD and premature death) is still an important barrier to the effective management of the disease. SGLT2 inhibitors, which were originally developed to treat type 2 diabetes, have demonstrated protective cardio-renal benefits and may aid in weight loss without causing marked hypoglycemia. Despite their benefits, prescription of these agents is still low, even among eligible at-risk patients [83]. Numerous RCTs evaluated the effects of SGLT2 inhibitors with regard to the improvement of cardio-renal outcomes in patients with diabetes and CKD, and SGLT2 inhibitors have quickly become the standard of care for DKD to delay progression to ESKD, even when the reduction in GFR limits their antihyperglycemic effect. Because of the extraordinary results shown in the prespecified analysis of the DAPA CKD study, dapagliflozin has recently been approved in the European Union for the treatment of CKD in adults with and without type 2 diabetes (https://www.ema.europa.eu/en/medicines/human/EPAR/forxiga). Accordingly, future guidelines will likely recommend the use of SGLT2 inhibitors also in CKD patients without type 2 diabetes. The future challenge for clinical research will be to identify the best combination of SGLT2 inhibitors with other traditional (dietary protein restriction and RAAS inhibitors) and non-traditional (nonsteroidal MRA and ERA) nephroprotective interventions. Ongoing trials will shed light on the effectiveness of the combination of SGLT2 inhibitors with the MRA, Finerenone (CONFIDENCE study-NCT05254002), and the ERA, Zibotentan (ZENITH study- NCT04724837).
